# Not all digital media use is equal: parental involvement moderates the association between children’s digital media use and parental stress

**DOI:** 10.3389/fpsyg.2026.1885062

**Published:** 2026-08-18

**Authors:** Weimiao Wang, Yuke Li, Yafei Lu, Yanjin Liu, Mohd Hanafi Mohd Yasin

**Affiliations:** 1 Department of Infant and Toddler Development and Health Management, School of Early Childhood Education, Shaanxi Xueqian Normal University, Xi'an, China; 2 Faculty of Education and Liberal Arts, INTI International University, Nilai, Malaysia

**Keywords:** children’s digital media use, device type, parental involvement, parental stress, preschool children

## Abstract

**Introduction:**

Digital media has become increasingly integrated into children’s daily lives. Although parents are often concerned about their children’s excessive use of digital media, digital devices may also be used as a coping strategy for parents experiencing stress. Furthermore, parental involvement in children’s digital media use has been found to be associated with parental stress. The present study examined the moderating role of parental involvement in the association between parental stress and children’s digital media use.

**Methods:**

A total of 674 parents of preschool children aged 3 to 6 years in China participated in this study. Parents reported their levels of parenting stress, their involvement in children’s digital media use, and their children’s digital media use.

**Results:**

Results indicated that the positive association between parental stress and screen time was weaker at higher levels of parental involvement. Similarly, the positive association between parental stress and children’s use of mobile digital devices, particularly smartphones, was weaker at higher levels of parental involvement. In contrast, no significant moderation pattern was observed for traditional media use, such as television. Emerging digital devices, including smart speakers, virtual reality devices, and smartwatches, showed a distinct pattern. At high levels of parental involvement, children’s use of emerging digital devices was positively associated with parental stress, whereas at low levels of parental involvement, this association was negative. Furthermore, parental involvement did not significantly moderate the associations between different media-use purposes and parental stress.

## 1 Introduction

In the past few years, concerns have been widely raised about children’s early and prolonged exposure to digital media (Hu et al., 2020; Vedechkina and Borgonovi, 2021; Chaarani et al., 2022; Jing et al., 2023). Building on these concerns, some researchers warn that excessive digital media use in early child development may have serious implications for cognitive and developmental outcomes. Notably, it is associated with obesity (Poitras et al., 2017), lower levels of physical activity (Shetty et al., 2022), poorer sleep quality (Lissak, 2018), language difficulties (Hill et al., 2016), and visual health problems (French et al., 2013; Yang et al., 2020) in young children, potentially damaging their physical development. Furthermore, young children with excessive screen time are at increased risk of mental health problems related to attention (Bauerlein and Walesh, 2009), anxiety (Nakshine et al., 2022), emotional regulation (Hu et al., 2020), and executive function (Corkin et al., 2021). At the same time, digital media use is not always harmful. When used appropriately, digital devices may support aspects of preschool children’s cognitive (Chaarani et al., 2022), language (Madigan et al., 2020), socio-emotional (Hu et al., 2020), and physical development (Aghanyan, 2023). Therefore, the main issue is not the use of digital media in general, but rather how children use it.

Parents are increasingly concerned about the potential adverse effects of children’s digital media use (McDaniel and Radesky, 2020). For example, a quantitative survey found that 71% of parents are concerned about children spending excessive screen time (Auxier et al., 2020). Despite these concerns, parents have contradictory attitudes toward screen use. On the one hand, they worry about the adverse influence of digital media, while on the other hand, they also use it to relieve their own stress (Ly et al., 2026). Kerr et al. (2025) further show that family media use can take on regulatory or relational functions depending on parents’ emotional states, suggesting that parental involvement is an important context for understanding how children’s digital media use is associated with parental stress.

Parental involvement plays an important role in shaping children’s use of digital media (Wu et al., 2026). Çalhan and Göksu (2024) found that parental role modeling and involvement were associated with children’s digital media use and game addiction, suggesting that parental involvement may help structure media use and reduce the likelihood of problematic patterns. Emerging research suggests that it may also be associated with parents’ psychological well-being, particularly in relation to parental stress (Williams et al., 2020). Parental involvement may influence parental stress indirectly by enhancing parents’ sense of efficacy (Kestler-Peleg et al., 2020).

The present study examined the moderating role of parental involvement in the association between parental stress and children’s digital media use. By examining screen time, device type, and usage purpose together, this study offers an integrated framework for exploring the associations between digital environments and parental stress.

### 1.1 Children’s digital media engagement and parental stress

The association between children’s digital media use and parental stress is widely described as bidirectional (Ly et al., 2026). Studies suggest that increased screen time among children is associated with higher parenting stress, possibly reflecting parental concerns about the risks of excessive screen use (Domoff et al., 2019). Parents report associations between excessive screen time and reduced social interaction, increased externalizing behaviors such as aggression or hyperactivity, increased emotional distress, and reduced creativity (Mallawaarachchi et al., 2022; McDaniel and Radesky, 2020). Efforts to restrict children’s screen use may also create tension and increase parent–child conflict (Hiniker et al., 2016; Coyne et al., 2017; Wolfers et al., 2025).

In turn, parenting stress has been linked to greater dependence on digital media in children. Under stress, parents may be more likely to allow or promote digital media use to manage caregiving demands or secure time to rest (Radesky et al., 2015; Nikken and Schols, 2015; Hiniker et al., 2016). This trend may be particularly relevant among parents of preschool children, as this developmental period often involves frequent caregiving demands related to behavioral guidance, emotional regulation, and daily supervision (Holly et al., 2019). In support of this pattern, prior research indicates that children’s negative affectivity is correlated with higher maternal stress, which, in turn, may be associated with extended screen time for children (Shin et al., 2021).

### 1.2 Parental involvement in children’s digital media use

Parental involvement might moderate the association between children’s digital media use and parental stress. In the context of digital media, parental involvement refers to strategies parents use to manage children’s digital media use and address potential risks; this is also known as parental mediation (Yao et al., 2022; Benedetto and Ingrassia, 2021; Nikken and Jansz, 2014). In the present study, the broader term parental involvement is used to refer collectively to these practices. Parental involvement may be associated with more positive patterns of children’s digital media engagement. For example, parents may guide children’s responsible technology use, which may be associated with lower levels of problematic use (Çalhan and Göksu, 2024). Longitudinal evidence suggests that parental involvement at age 3 predicted lower screen time 1 year later (Fitzpatrick et al., 2023). Additionally, parental involvement can support learning by helping children review, practice, and expand on school-based knowledge (Şad et al., 2016).

Parental involvement may also be associated with lower parental stress. For example, Williams et al. (2020) found that highly engaged parents reported lower parenting stress than less engaged parents in a supported playgroup. Furthermore, active participation may be associated with a stronger sense of parental efficacy, which is related to parental stress according to self-efficacy theory (Park and Oh, 2023; Bandura, 1977). These findings suggest that parental involvement may moderate the association between children’s digital media use and parental stress and may be relevant to children’s responsible digital media use.

### 1.3 Theoretical framework

The Parenting Stress Theory views parental stress as a normal part of everyday caregiving. According to Abidin’s Parent–Child Relationship model, parental stress stems from parent characteristics, child characteristics, and parent–child interactions (Abidin, 1995). Parents may need to determine an appropriate screen time for their children, evaluate the suitability of the content, address their children’s resistance, and enforce rules regarding device access. These demands may be related to increased parental stress. This framework explains the relationship between parental stress and children’s use of digital media.

The Stress and Coping Theory explains how parents manage these demands. According to Lazarus and Folkman (1984), stress is shaped not only by the presence of a demand, but also by how individuals respond to it. Parental involvement in children’s digital media use can be understood as a related coping process. By setting rules, providing explanations, and using media together with their children, parents may organize their children’s media experiences and manage the associated pressures. In this sense, parental involvement may be related to differences in how children’s digital media use is associated with parental stress.

The Techno-Microsystem Framework further emphasizes that children’s digital media use is embedded in family environments and parent–child routines (Johnson, 2010). Specifically, digital media use is conceptualized as a contextual factor within the family system. In other words, different forms of media use may create different caregiving conditions. For example, Television is often used in shared family spaces and may be easier for parents to observe. Smartphones and tablets are more portable and individualized, which may make supervision more difficult. These differences suggest that screen time, device type, and purpose of use may relate to parental stress in distinct ways.

By integrating these perspectives, the present study conceptualizes preschool children’s digital media use as a media-related caregiving context within the family system. Parenting Stress Theory explains why this context may be linked to parental stress. Stress and Coping Theory explains why parental involvement may shape parents’ responses to media-related demands. The Techno-Microsystem Framework explains why these associations may vary across dimensions of media use. Based on this framework, the study examined parental involvement as a moderator of the associations between children’s digital media use and parental stress across screen time, device type, and purpose of use.

### 1.4 The present study

Researchers have studied children’s digital media use, but how parental involvement is associated with such use remains unclear, especially in early childhood. Few researchers have considered family dynamics, such as parental involvement, that may be relevant to this association. Many studies also treat digital media use as total screen time, ignoring differences in device type and purpose.

**Figure 1:**
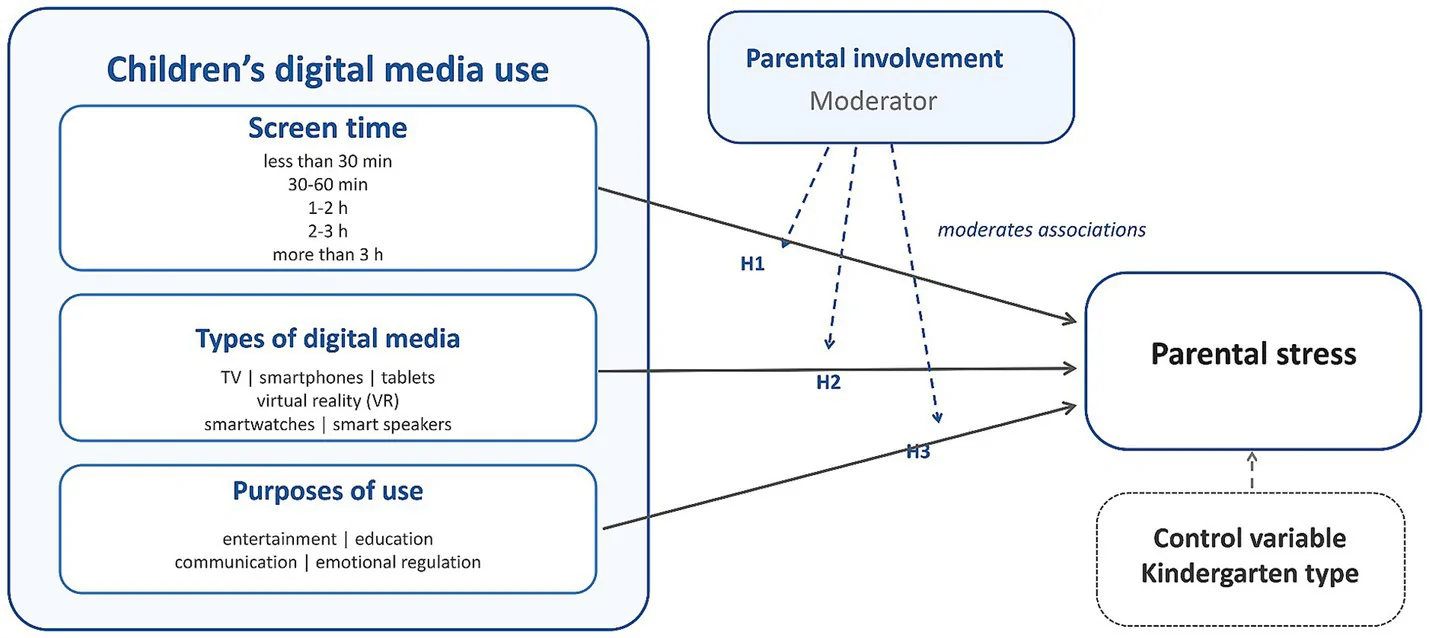
Conceptual moderated model.

To address this gap, we investigate how children’s digital media use is associated with parental stress, with a specific focus on parental involvement as a moderating factor (see Figure 1). Building on the above framework and evidence, we propose the following hypotheses:

Prospective evidence indicates that parental mediation at age 3 is associated with reduced screen time after 1 year (Fitzpatrick et al., 2023). Accumulating evidence has shown a positive correlation between parental stress and screen time (Ly et al., 2026; McDaniel and Radesky, 2020; Brauchli et al., 2024). Consequently, this study examined the moderating role of parental involvement in the association between children’s screen time and parental stress.

*Hypothesis 1*: Parental involvement will moderate the association between parental stress and children’s screen time. As parental involvement increases, the positive association between screen time and parental stress will become progressively weaker.

The association between parental stress and children’s digital media use may differ by device type. Parenting stress may be more closely associated with children’s use of interactive and mobile media, such as tablets and digital games, than with traditional television use (McDaniel and Radesky, 2020). Consequently, this study further examines whether parental involvement moderates the association between children’s use of various digital devices (e.g., television, smartphones, tablets, and emerging devices) and parental stress.

*Hypothesis 2*: Parental involvement will moderate the relationship between parental stress and children’s use of mobile digital products, such as tablets and smartphones. In contrast, parental involvement is not anticipated to significantly moderate the association between parental stress and the use of traditional or other digital devices. As parental involvement increases, the positive association between parental stress and children’s use of mobile digital products is expected to be weaker.

The purposes of digital media use include entertainment, education, communication, and emotional regulation (Chaudron et al., 2015; Radesky et al., 2015; Marsh et al., 2016; Papadakis et al., 2018a, 2018b). The association between parental stress and children’s digital media use may also differ by purpose of use. When parents are under pressure, screens are more likely to be seen as tools for soothing emotions and entertainment (Kattein et al., 2023). However, parental stress shows little association with educational and communication purposes (Benedetto and Ingrassia, 2021). Consequently, this study further examines whether parental involvement moderates the association between children’s purposes of digital media use (e.g., entertainment, education, communication, and emotional regulation) and parental stress.

*Hypothesis 3*: Parental involvement will moderate the association between parental stress and children’s use of digital devices for entertainment and emotional regulation, whereas its role in communication and education is not expected to be significant. As parental involvement increases, the positive association between entertainment use and parental stress will become progressively weaker. As levels of parental involvement increase, the positive association between emotional regulation use and parental stress will become progressively weaker.

## 2 Methodology

### 2.1 Sample

A sample of 700 parents of preschool children was recruited from three types of public kindergartens in Xi’an, Shaanxi Province, China. The three categories were Shaanxi Provincial Model Kindergartens, the highest officially recognized category in the local system; Xi’an Municipal Model Kindergartens; and ordinary public kindergartens. A multistage sampling strategy was adopted. One kindergarten was selected from each category. Within each participating kindergarten, six classes were randomly selected, with two from each age group (3–4, 4–5, and 5–6 years). Parents of children in the selected classes were invited to complete the questionnaire. The inclusion criteria were parents of preschool children aged 3–6 years enrolled in the selected kindergartens who provided informed consent.

After incomplete or invalid responses were removed, 674 questionnaires were retained for analysis, yielding a valid response rate of 96.29%. Invalid responses included patterned answering, such as giving the same response across questionnaire items. Among the children included in the final sample, 29.7% were aged 3–4 years, 33.1% were aged 4–5 years, and 37.2% were aged 5–6 years. Most parents were 35–40 years old (56.2%). In terms of educational attainment, 51.5% of parents held a bachelor’s degree, 39.0% had a junior college education or lower, and 9.5% held a master’s degree or higher (see Table 1).

**Table 1:** Demographic profile of study respondents.

Variable	*n*	%
Kindergarten of child		
Provincial Model Kindergarten	230	34.1
Xi’an Municipal Model Kindergartens	225	33.4
Ordinary Public Kindergartens	219	32.5
Child Gender		
Male	355	52.7
Female	319	47.3
Age of children		
3–4	200	29.7
4–5	223	33.1
5–6	251	37.2
Parents Gender		
Male	102	15.1
Female	572	84.9
Age of parents		
25–30	21	3.1
30–35	260	38.6
35–40	379	56.2
40–45	14	2.1
Education of parents		
Junior college and below	263	39.0
Bachelor degree	347	51.5
Master’s degree and above	64	9.5

### 2.2 Measures

#### 2.2.1 Parenting stress

This study used the Chinese revised version of Abidin’s Parenting Stress Index–Short Form (PSI-SF). It includes 36 items across three dimensions: parental distress (e.g., “I have given up much of the life I had looked forward to because of my child”), parent–child dysfunctional interaction (e.g., “My child rarely does things that make me feel happy”), and difficult child (e.g., “My child has more problems than I expected”). All items were scored on a 5-point scale (1 = strongly disagree, 5 = strongly agree). Item scores were summed to generate a total parental stress score, with higher scores indicating greater parental stress. In this study, Cronbach’s alpha coefficients were 0.920, 0.928, and 0.882 for parental distress, interaction, and difficult child, respectively.

#### 2.2.2 Children’s digital media use

Children’s screen time was assessed using a single estimate to reduce respondent burden. This approach is often used in large-sample surveys to balance measurement with questionnaire length and completion rates (Wolfers et al., 2025; Zhang, 2025; Wu et al., 2026). Parents were asked to report how much time their child typically spent using digital media each day. The response options were: less than 30 min; 30–60 min; 1–2 h; 2–3 h; and more than 3 h. Parents selected only one response option. Device type was assessed using a multiple-response item to capture the range of digital devices preschool children use. Parents indicated whether their child used each of the following devices: television, smartphone, tablet, virtual reality (VR) device, smartwatch, and smart speaker. Each device was coded as a binary variable for analysis: 0 indicated the device was not selected, and 1 indicated it was selected. Four primary purposes of children’s digital media use were assessed based on prior research: entertainment, education, communication, and emotional regulation (Chaudron et al., 2015; Radesky et al., 2015; Marsh et al., 2016; Papadakis et al., 2018a, 2018b). Each purpose was coded as a binary indicator for the analyses (0 = not selected, 1 = selected).

#### 2.2.3 Parental involvement

The Parental Media Mediation Scale was used to assess parental involvement in children’s digital media use (Nikken and Jansz, 2014). Similar parental mediation constructs have also been used in Chinese digital media research, with evidence of good reliability and validity (Lou et al., 2024). Previous work has conceptualized parental mediation as a multidimensional construct, including restrictive mediation, active mediation, co-use, and supervision. Sample items were “I restrict my child’s use of digital media in terms of time and location” for restrictive mediation, “I talk to my child about the benefits and harms of using digital media” for active mediation, “I check my child’s usage history after they use digital media” for supervision, and “I use digital media together with my child to search for learning materials” for co-use. In the present study, the scale consisted of 12 items covering four forms of parental mediation behavior. Each dimension included three items. Items were rated on a 5-point scale from 1 (strongly disagree) to 5 (strongly agree). Scores were summed across the 12 items to produce a total parental involvement score, with higher scores indicating greater involvement in children’s digital media use. Although the measure covered four domains of mediation behavior, the total score was used in the present analysis to represent overall parental involvement. The adapted scale showed good internal consistency in the current sample, Cronbach’s alpha = 0.837.

### 2.3 Analysis strategy

All analyses were conducted using SPSS version 26.0 and the PROCESS macro. Descriptive statistics were used to summarize children’s digital media use. Moderation analyses were conducted using the PROCESS macro (Model 1), based on multiple linear regression to examine the moderating role of parental involvement. Parental involvement was standardized (ZI), with 0 corresponding to the sample mean and 1 corresponding to one standard deviation above the mean. Thus, Johnson–Neyman values were interpreted in units of the standardized parental involvement score rather than raw questionnaire scores. The focal predictors and moderator were mean-centered before estimating the interaction terms. Kindergarten type was controlled using dummy variables. HC3 robust standard errors were used to address potential heteroscedasticity. Interaction coefficients, *R*^2^ values, and ΔR^2^ values were reported. Given the cross-sectional design, the results were interpreted as associations rather than causal effects.

To check for potential common method bias, Harman’s single-factor test was applied. All observed items were entered into an unrotated exploratory factor analysis, and the proportion of variance explained by the first factor was used as the diagnostic criterion.

## 3 Results

### 3.1 Descriptive statistics, correlations, and common method bias

Descriptive statistics of preschool children’s digital media use are presented in Table 2. Regarding usage time, most children used digital media for a relatively short time. Specifically, 34.87% of children used digital media for less than 30 min per day, while 44.66% used it for 30–60 min. A smaller proportion of children spent longer periods using digital media each day: 16.47% used it for 1–2 h, 2.08% for 2–3 h, and 1.92% for more than 3 h. Overall, more than 70% of children spent less than 1 h per day on digital media.

**Table 2:** Distribution of children’s digital media use.

Category	Frequency (*n*)	Percentage (%)
Usage time		
Less than 30 min	235	34.87
30–60 min	301	44.66
1–2 h	111	16.47
2–3 h	14	2.08
More than 3 h	13	1.92
Devices		
Television	522	77.40
Mobile phone	336	49.90
Tablet	287	42.63
Emerging digital devices	193	28.60
Purpose		
Entertainment	522	77.43
Educational	453	67.20
Communication	288	42.70
Emotional regulation	146	21.70

In terms of device type, television was the most commonly used device (77.40%), followed by mobile phones (49.90%) and tablets (42.63%). Emerging digital devices, such as smartwatches and smart speakers, were used by 28.60% of the children. Regarding purpose of use, entertainment was the most frequently reported (77.43%), followed by educational use (67.20%). Communication use was reported by 42.70% of parents, whereas emotional comfort was the least common purpose (21.70%).

The mean scores, SDs, maximums, and minimums for parental stress and parental involvement are shown in Table 3. Spearman’s correlation indicated a negative association between parental stress and parental involvement.

**Table 3:** Descriptive statistics and associations.

Variable	M	SD	Min	Max	1	2
Parental stress	78.32	18.97	39	142	–	−0.234^**^
Parental involvement	44.59	6.40	12	60	−0.234^**^	–

**p < 0.01.

The assessment of common method bias did not indicate a substantial single-factor structure. In the unrotated exploratory factor analysis, the first factor accounted for 29.63% of the total variance, below the commonly used 40% threshold for Harman’s single-factor test. Thus, common method bias was unlikely to pose a major threat to the interpretation of the observed associations.

### 3.2 Moderating role of screen time on parental stress

Using the PROCESS macro (Model 1; Hayes, 2018), kindergarten type was controlled as a contextual covariate through two dummy variables, with provincial demonstration kindergartens as the reference group. Parental involvement was entered as a standardized score. The model was statistically significant, *R*^2^ = 0.058, *F*(5, 674) = 8.295, *p* < 0.001. The interaction between screen time and parental involvement explained a small but statistically significant additional proportion of variance in parental stress, ΔR^2^ = 0.006, *F*(1, 674) = 4.189, *p* = 0.041. These findings indicate that the association between children’s screen time and parental stress differed by level of parental involvement, but the effect should be interpreted as a modest moderation rather than a strong predictive factor.

As shown in Table 4, longer screen time was positively associated with parental stress, *B* = 2.510, *p* = 0.002, 95% CI [0.920, 4.101]. The interaction term between screen time and parental involvement was also statistically significant, *B* = −1.893, *p* = 0.041, 95% CI [−3.710, −0.077].

**Table 4:** Moderation analysis predicting parental stress.

Predictor	*B*	*SE*	*t*	*p*	LLCI	ULCI
Constant	76.420	1.002	76.297	< 0.001	74.453	78.386
Screen time (X)	2.510	0.810	3.099	0.002	0.920	4.101
Parental Involvement(W)	−3.193	0.726	−4.396	< 0.001	−4.619	−1.767
X × W	−1.893	0.925	−2.047	0.041	−3.710	−0.077
Kindergarten type 2	2.017	2.416	0.835	0.404	−2.727	6.761
Kindergarten type 3	4.202	1.514	2.776	0.006	1.230	7.174

B = unstandardized coefficients; SE, standard error; CI, confidence interval. X = children’s screen time; W = parental involvement. Parental involvement was entered as a standardized score (ZI). The focal predictor and moderator were mean-centered before estimating the interaction term. Kindergarten type was controlled using dummy variables. HC3 robust standard errors were used.

**Figure 2:**
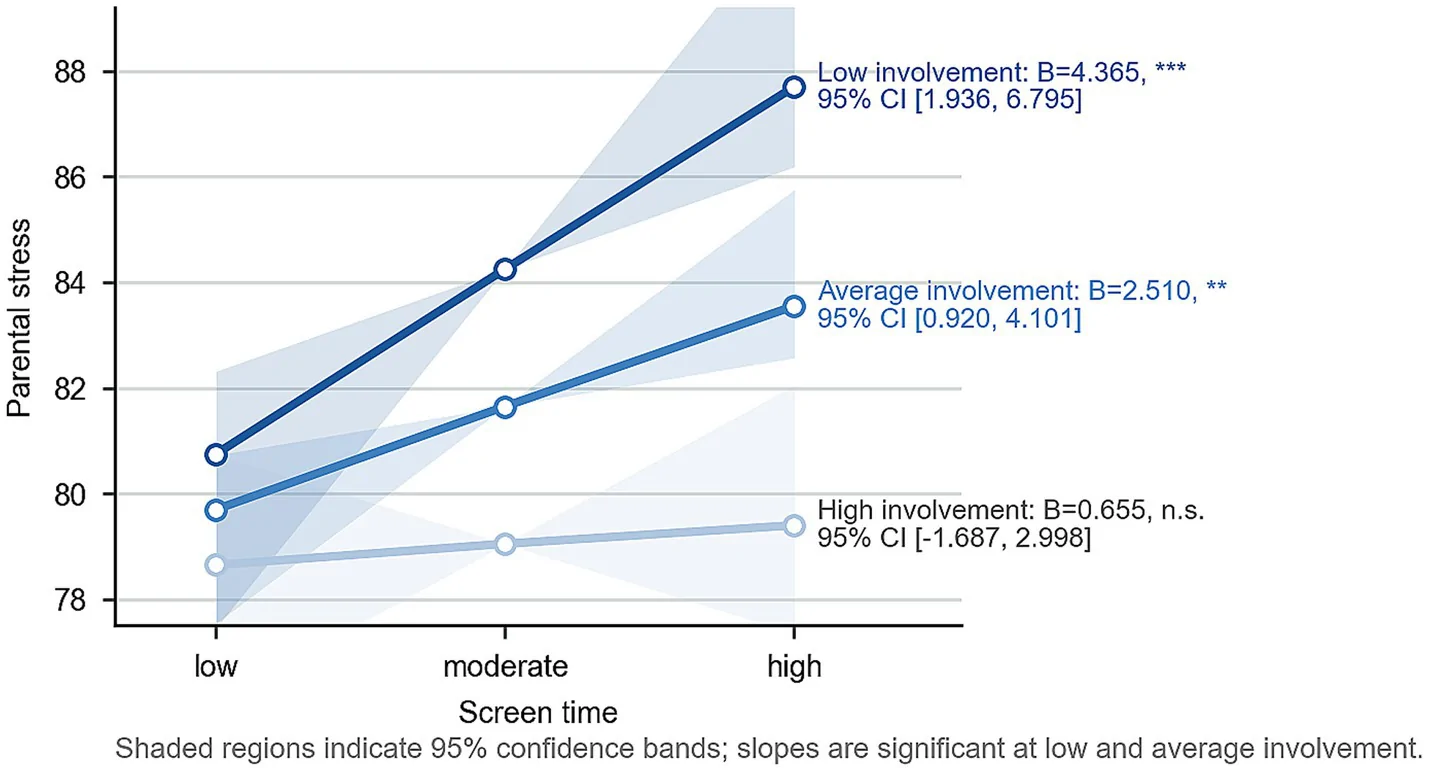
Simple slope plot.

Simple slope analyses further showed this pattern. Screen time was positively associated with parental stress when parental involvement was low (−1 SD), *B* = 4.365, *p* < 0.001, 95% CI [1.936, 6.795], and at the mean level, *B* = 2.510, *p* = 0.002, 95% CI [0.920, 4.101]. In contrast, the association was not statistically significant when parental involvement was high (+1 SD), *B* = 0.655, *p* = 0.583, 95% CI [−1.687, 2.998] (see Figure 2).

**Figure 3:**
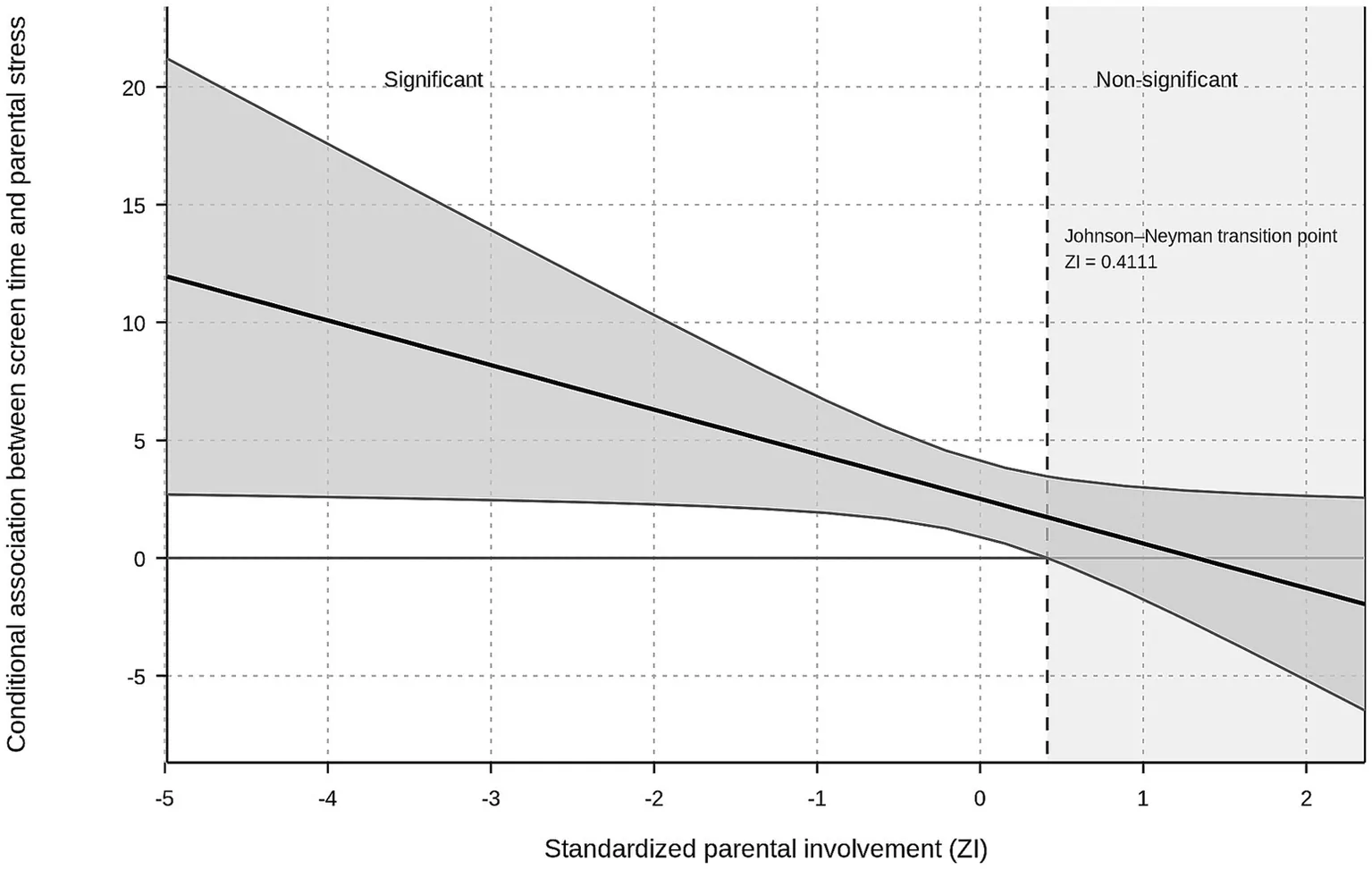
LOOP plot of the conditional association between children’s screen time and parental stress across levels of parental involvement.

The Johnson-Neyman analysis indicated that screen time was positively associated with parental stress when standardized parental involvement scores were below 0.411. As shown in Figure 3, when parental involvement exceeded this threshold, the association between screen time and parental stress was no longer statistically significant (see Table 5).

**Table 5:** Conditional effects of time on parental stress at different levels of involvement.

Parental involvement level	Effect (B)	SE	*t*	*p*	95% CI lower	95% CI upper
Low	4.365	1.237	3.528	<0.001	1.936	6.795
Average	2.510	0.810	3.099	0.002	0.920	4.101
High	0.655	1.193	0.549	0.583	−1.687	2.998

Low, average, and high parental involvement refer to one standard deviation below the mean, the mean, and one standard deviation above the mean, respectively. Values are based on the standardized parental involvement score (ZI), where 0 represents the sample mean and 1 represents one standard deviation above the mean.

### 3.3 Moderating effect of digital media type on parental stress

To examine whether parental involvement moderated the association between device-specific screen use and parental stress, separate moderation models were estimated for television, smartphone, tablet, and emerging digital device use, controlling for kindergarten type. All four models were statistically significant, although their explanatory power was modest, with *R*^2^ values ranging from 0.054 to 0.083. Significant interaction effects were found for smartphone use, ΔR^2^ = 0.0093, *F*(1, 674) = 5.26, *p* = 0.022, and emerging digital device use, ΔR^2^ = 0.019, *F*(1, 674) = 7.79, *p* = 0.005. The interaction between tablet use and parental involvement was marginally significant, ΔR^2^ = 0.0065, *F*(1, 674) = 3.35, *p* = 0.068. In contrast, the interaction effect was not significant for television use, ΔR^2^ = 0.0007, *F*(1, 674) = 0.30, *p* = 0.584. Although some interaction terms were statistically significant, the models’ explanatory power was modest, with R^2^ values ranging from 0.054 to 0.083.

Specifically, after controlling for kindergarten type, smartphone use (*B* = 5.637, *p* < 0.001, 95% CI [3.332, 7.943]) and tablet use (*B* = 4.859, *p* < 0.001, 95% CI [2.590, 7.128]) showed positive main associations with parental stress. In contrast, television use (*B* = 1.810, *p* = 0.296, 95% CI [−1.589, 5.209]) and emerging digital device use (*B* = −0.186, *p* = 0.911, 95% CI [−3.445, 3.072]) were not significantly associated with parental stress. For the interaction terms, the smartphone use × parental involvement interaction was negative and statistically significant (*B* = −3.240, *p* = 0.022, 95% CI [−6.015, −0.465]). In contrast, the emerging digital device use × parental involvement interaction was positive and statistically significant (*B* = 6.119, *p* = 0.005, 95% CI [1.815, 10.423]). The interaction for tablet use was negative but only marginally significant (*B* = −2.643, *p* = 0.068, 95% CI [−5.478, 0.192]), whereas the interaction for television use was not significant (*B* = 1.262, *p* = 0.584, 95% CI [−3.262, 5.785]) (see Table 6).

**Table 6:** Moderation analyses of device type and parental involvement in predicting parental stress.

Predictor (X)	Interaction term	B	SE	*t*	*p*	*R* ^2^	ΔR^2^	95% CI
Television	TV × Parental involvement	1.262	2.304	0.548	0.584	0.054	0.001	[−3.262, 5.785]
Smartphone	Smartphone × parental involvement	−3.240	1.413	−2.293	0.022^*^	0.083	0.009	[−6.015, −0.465]
Tablet	Tablet × Parental involvement	−2.643	1.444	−1.830	0.068	0.076	0.007	[−5.478, 0.192]
Emerging digital devices	Emerging devices × parental involvement	6.119	2.192	2.792	0.005^**^	0.063	0.019	[1.815, 10.423]

B = unstandardized coefficient; SE, standard error. *R*^2^ refers to the variance explained by the full model, and ΔR^2^ refers to the change in R^2^ attributable to the interaction term. Johnson–Neyman values are reported in units of the standardized parental involvement score (ZI), where 0 represents the sample mean and 1 represents one standard deviation above the mean. These values should not be interpreted as raw questionnaire scores. Kindergarten type was controlled using dummy variables. HC3 robust standard errors were used. ^*^p < 0.05, ^**^p < 0.01.

Simple slope analyses further showed this pattern. As shown in Table 7, at low (*B* = 8.688, *p* < 0.001, 95% CI [5.335, 12.042]) and mean (*B* = 5.637, *p* < 0.001, 95% CI [3.332, 7.943]) levels of parental involvement, smartphone use among preschool children showed a positive association with parental stress. However, this association was not statistically significant at high levels of parental involvement (*B* = 2.586, *p* = 0.160, 95% CI [−1.025, 6.197]). The pattern for emerging digital devices differed. When parental involvement was high (*B* = 5.692, *p* = 0.034, 95% CI [0.446, 10.937]), emerging digital media use was significantly and positively associated with parental stress. Conversely, when parental involvement was low (*B* = −6.064, *p* = 0.025, 95% CI [−11.347, −0.782]), preschool children’s use of emerging digital media was negatively associated with stress levels. At mean levels of parental involvement, this association was not significant (*B* = −0.186, *p* = 0.911, 95% CI [−3.445, 3.072]).

**Table 7:** Conditional effects of different digital devices on parental stress at different levels of involvement.

Independent variable	Parental involvement	Effect (B)	SE	*t*	*p*	95% CI
Smartphone	Low	8.688	1.708	5.087	<0.001^***^	[5.335, 12.042]
	Average	5.637	1.174	4.801	<0.001^***^	[3.332, 7.943]
	High	2.586	1.839	1.406	0.160	[−1.025, 6.197]
Emerging digital device	Low	−6.064	2.690	−2.254	0.025^*^	[−11.347, −0.782]
	Average	−0.186	1.660	−0.112	0.911	[−3.445, 3.072]
	High	5.692	2.672	2.130	0.034^*^	[0.446, 10.937]

Low, average, and high parental involvement refer to one standard deviation below the mean, the mean, and one standard deviation above the mean of the standardized parental involvement score (ZI), respectively. Values are based on the standardized parental involvement score (ZI), where 0 represents the sample mean and 1 represents one standard deviation above the mean. These values should not be interpreted as raw questionnaire scores.

**Figure 4:**
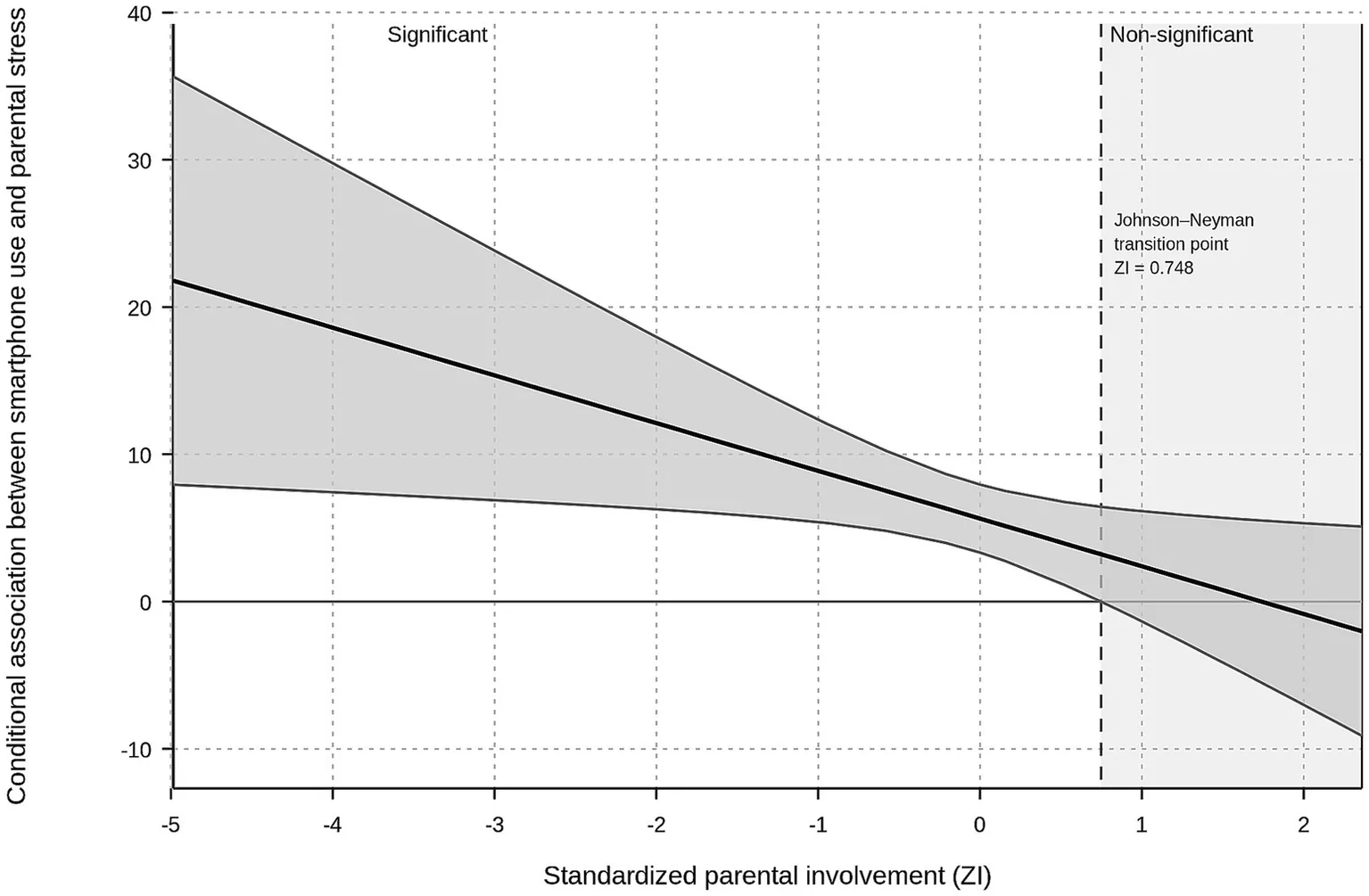
LOOP plot of the conditional difference in parental stress by children’s smartphone use across levels of parental involvement.

**Figure 5:**
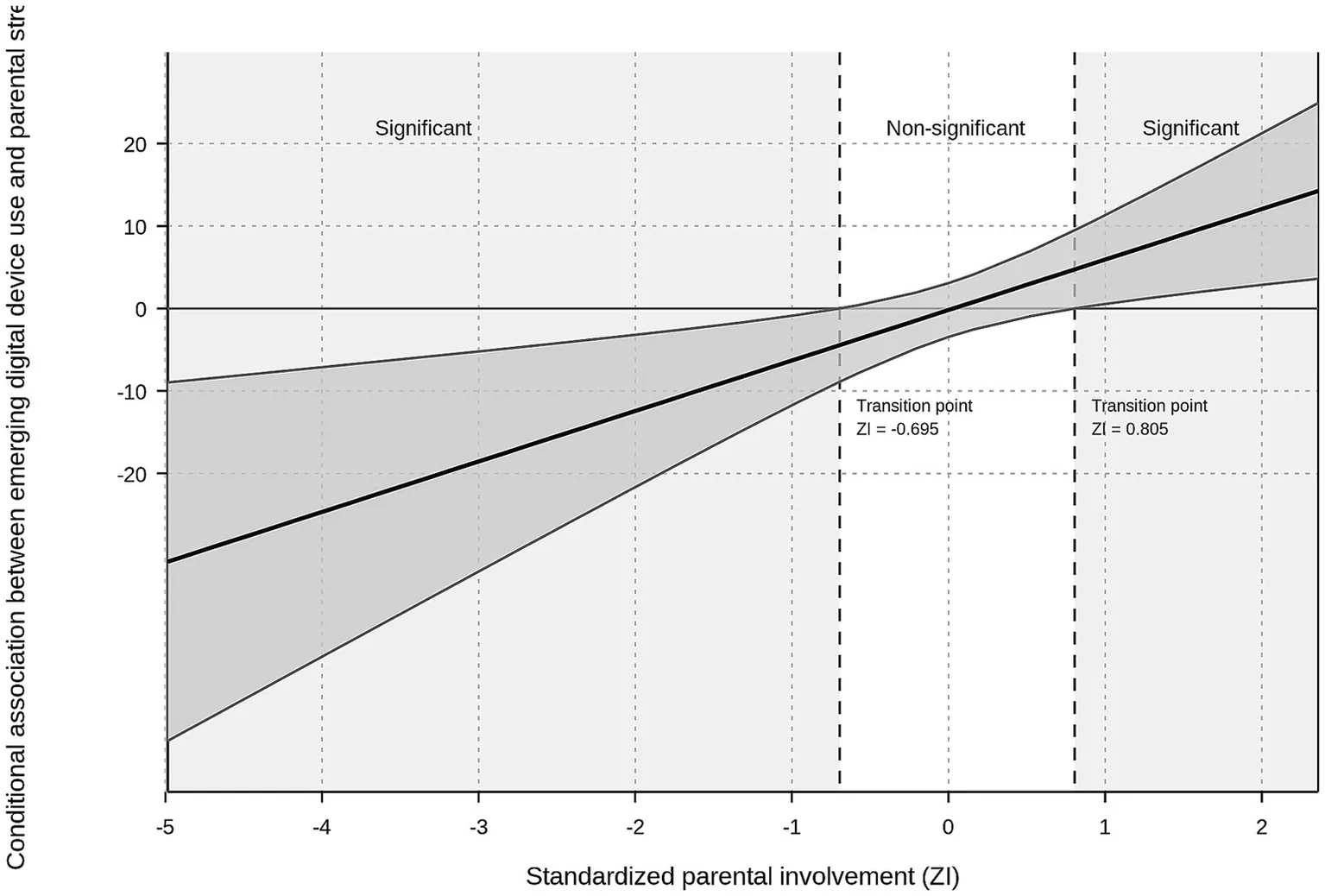
LOOP plot of the conditional difference in parental stress by children’s emerging digital device use across levels of parental involvement.

As shown in Figure 4, Johnson-Neyman analyses further showed that smartphone use was positively significantly associated with parental stress when parental involvement scores were below 0.748. As shown in Figure 5, the association between emerging digital devices and parental stress was significant when parental involvement scores were below −0.695 or above 0.805.

### 3.4 Moderating effect of media use purpose on parental stress

To examine whether parental involvement moderated the association between purposes of digital media use and parental stress, we compared different purposes, including entertainment, communication, emotional regulation, and education, while controlling for kindergarten type. The overall models were statistically significant, but their explanatory power was modest, with *R*^2^ values ranging from 0.046 to 0.061. No significant interaction effects were found for entertainment use, ΔR^2^ = 0.0006, *F*(1, 674) = 0.25, *p* = 0.615; educational use, ΔR^2^ = 0.001, *F*(1, 661) = 0.38, *p* = 0.536; communication use, ΔR^2^ = 0.0001, *F*(1, 674) = 0.04, *p* = 0.833; or emotional regulation use, ΔR^2^ = 0.0016, *F*(1, 674) = 0.89, *p* = 0.347. Overall, the analyses did not show evidence that parental involvement significantly moderated the associations between children’s purposes of digital media use and parental stress (see Table 8).

**Table 8:** Moderation analyses of purpose and parental involvement in predicting parental stress.

Predictor (X)	Interaction term	B	SE	*t*	*p*	ΔR^2^	*R* ^2^	95% CI
Communication	Communication × parental involvement	−0.368	1.746	−0.211	0.833	0.000	0.046	[−3.796, 3.060]
Emotional regulation	Emotional regulation × parental involvement	−1.703	1.809	−0.941	0.347	0.002	0.061	[−5.255, 1.850]
Educational	Educational × parental involvement	0.994	1.605	0.619	0.536	0.001	0.046	[−2.159, 4.147]
Entertainment	Entertainment × parental involvement	−0.906	1.798	−0.504	0.615	0.001	0.046	[−4.437, 2.626]

B = unstandardized coefficient; SE, standard error. *R*^2^ refers to the variance explained by the full model, and ΔR^2^ refers to the change in *R*^2^ attributable to the interaction term. Parental involvement was standardized and mean-centered before creating the interaction terms. Kindergarten type was controlled using dummy variables. HC3 robust standard errors were used.

## 4 Discussion

This study presents three main findings. First, parental involvement was found to moderate the association between preschoolers’ screen time and parental stress. Specifically, the positive association between screen time and parental stress was weaker among families with higher levels of parental involvement than among those with lower levels of parental involvement.

Second, parental involvement also moderated the association between preschoolers’ use of different types of digital media (e.g., television, smartphones, tablets, and emerging devices) and parental stress. Notably, this moderation pattern varied across media types. For smartphones, the positive association with parental stress was weaker at higher levels of parental involvement. In contrast, for emerging digital devices, the association with parental stress differed by levels of parental involvement. More specifically, the association between parental stress and the use of emerging devices was significantly positive at high levels of parental involvement but significantly negative at lower levels.

Finally, parental involvement did not appear to moderate the association between different media-use purposes (e.g., entertainment, education, emotional regulation, and communication) and parental stress, suggesting that parental involvement’s moderation pattern may be more evident for the form of media use than for its intended purpose.

### 4.1 Parental involvement weakens the association between screen time and parental stress

The results suggest that parental involvement moderated the association between preschoolers’ screen time and parental stress. Specifically, the positive association was weaker at higher levels of parental involvement. This moderation pattern can be understood through the P-C-R model of parental stress (Abidin, 1995). The Parent–Child-Relationship model proposes that parental stress is shaped by parent-related factors, child-related factors, and the quality of parent–child interaction (Abidin, 1995).

The P-C-R model provides a useful framework for interpreting this finding. Longer screen time may be associated with higher parental stress, possibly reflecting disruptions in everyday parent–child interactions. Prior research has identified a negative association between parenting stress and the quality of parent–child relationships. Higher levels of stress are associated with lower quality of the parent–child relationship (Yu S. et al., 2025). By fostering interaction and communication, parental involvement may be associated with lower levels of media-related stress. For example, Lapierre et al. (2021) emphasized the important role of family communication in relation to both children’s digital media use and family stress. Similarly, Bayar et al. (2026) also reported that active parental involvement in preschool children’s digital media use is associated with improvements in the quality of parent–child relationships. Collectively, these findings suggest that involvement may partly explain why the association between screen time and parental stress was weaker among families with higher-quality relationships.

In addition, parental involvement may operate by influencing the parental characteristics in the P-C-R model. Active involvement may be associated with higher parental efficacy in managing children’s behaviors and daily activities. Empirical evidence suggests that parents who actively engage in their children’s lives report significantly higher parenting efficacy (Benson, 2015; Glatz and Buchanan, 2015). From a self-efficacy perspective, increased perceived parenting efficacy may help parents manage stress more effectively (Rezendes and Scarpa, 2011; Park and Oh, 2023; Bandura, 1977). Specifically, parenting efficacy is negatively correlated with levels of parenting stress (Lee et al., 2023). In line with this reasoning, parental involvement may function not only as a behavioral strategy but also as an important psychological resource related to lower parental stress.

Finally, parental involvement may also be related to children’s patterns of digital media use. Accumulating evidence suggests that children’s psychosocial difficulties, including both externalizing and internalizing problems, are positively associated with higher parenting stress (Barroso et al., 2018; Operto et al., 2021; Lee et al., 2022; Fang et al., 2024). In this context, parental involvement can play a protective role (Çalhan and Göksu, 2024). Parental involvement can help children derive benefits from the digital world while reducing its potential adverse outcomes (Lin et al., 2025; Wu et al., 2026). Conversely, when parental involvement is limited and digital media may serve as a form of caregiving, children may be more likely to develop problematic patterns (Wang et al., 2024).

### 4.2 The moderating role of parental involvement differs across digital media types

This study examined how various forms of preschool children’s digital media use were associated with parental stress, with emphasis on the moderating role of parental involvement. The findings suggest that the associations varied by media type. Specifically, parental involvement significantly moderated the associations between smartphone use and parental stress, as well as between the use of emerging digital devices and parental stress. However, such moderation patterns were not observed for television. In addition, neither television use nor emerging digital media use showed a significant main association with parental stress, indicating that different forms of digital media were not associated with parental stress in the same way.

One possible explanation for these differences is how parents perceive various types of digital media. Parents’ attitudes regarding digital media play a significant role in influencing children’s media use. For example, when parents hold favorable attitudes toward digital media, children are more likely to spend more time on screens (Lee et al., 2022; Attavar and Rani, 2025). Furthermore, parental attitudes may also be related to parental stress. Evidence shows that parental attitudes may modify the relationship between parental stress and children’s screen use. In particular, more positive parental attitudes may strengthen the positive association between parental stress and children’s screen use (Brauchli et al., 2024).

Parents may hold different attitudes toward different types of digital media. Television is often regarded as a relatively safe and acceptable form of digital media for parents. Many parents use television to calm their children, allowing them to attend to household tasks or take short breaks (Bentley et al., 2016). In this context, parents may hold a more permissive attitude toward television use, as they view its practical caregiving benefits as outweighing potential risks (Sajjan, 2013; Chong et al., 2023).

In contrast, parents’ attitudes toward mobile devices are often mixed. Although these devices may be perceived as supporting learning and everyday caregiving, parents remain concerned about excessive use and dependency (Mallawaarachchi et al., 2022). Compared with mobile media, parents may be more receptive to emerging digital devices. Recent evidence from Nandhini et al. (2025) indicates that parents are generally receptive to the use of AI in early childhood education. According to this study, 74.4% of parents reported favorable attitudes regarding AI use in kindergartens, and 69.3% considered AI tools suitable for preschool children (Lin et al., 2025; Su, 2025). Parents perceive these technologies as caregiving tools. For example, smart speakers can provide parents with brief relief by telling stories and answering children’s questions (Mascheroni, 2024).

However, parental attitudes may not fully account for the moderating effects observed in this study. This finding can be interpreted through the Techno-Microsystem Framework, which incorporates digital technologies into children’s everyday family environments (Johnson, 2010). From this perspective, the associations between device use and parental stress may depend not only on device characteristics but also on how they are used in family routines.

As a traditional medium, television is relatively stable and predictable, making it easier for parents to monitor and regulate (Njoroge et al., 2013). In addition, television is used in shared family spaces, making co-viewing easier for parents (Warren et al., 2002). This may explain the nonsignificant association between television use and parental stress.

In contrast, smartphones and tablets are portable devices that enable children to access a wide variety of content in different situations. Although this flexibility may be beneficial in some situations, it may also complicate parental supervision and management (Bentley et al., 2016). Thus, moderate parental involvement may not be associated with lower parental stress concerning their children’s mobile media use. This may explain why mobile media use remains positively associated with parental stress at low and moderate levels of parental involvement.

Emerging digital devices showed a different pattern from television and mobile devices. The present study found that, at low levels of parental involvement, the use of emerging digital media was negatively associated with parental stress, whereas at high levels of parental involvement, this association was positive. This pattern suggests that the association between emerging digital media use and parental stress may depend on the level of parental involvement.

One possible explanation is that emerging digital devices may offer practical assistance with everyday caregiving tasks. When parental involvement is low, such devices may partially serve as temporary caregiving tools. For example, by telling stories and responding to children’s questions, emerging digital devices may be perceived as easing parents’ caregiving tasks (Mascheroni, 2024; Beneteau et al., 2020).

By contrast, greater parental involvement may make using emerging digital devices more challenging for parents. Instead of simply letting the device occupy their child, parents may need to learn how the technology works. For example, parents need to learn to monitor their child’s interactions and judge whether the content or responses are appropriate. These responsibilities may help explain why the use of emerging digital devices was not necessarily linked to lower parental stress among more involved parents. This interpretation aligns with recent concerns that parents must continually adapt to rapidly changing digital technologies and manage potential risks associated with AI-based systems (Widyaatmadja and Solechan, 2024; Yu Y. et al., 2025).

### 4.3 The limited role of parental involvement in moderating purpose-based media use

Unlike screen time and device type, the associations between purposes of digital media use and parental stress were not moderated by parental involvement. One possible explanation is that parents may not usually tailor their strategies to the purpose of children’s digital media use. Rather, they tend to manage children’s media use more generally. Existing research suggests that parents are inclined to regulate overall media use rather than distinguish between media use for learning and entertainment (Livingstone and Helsper, 2008). This may be related to the fact that, although parents often perceive digital media as supporting learning, children’s actual use is predominantly for entertainment (Tay et al., 2021).

In addition, preschool children’s media use often serves multiple purposes at the same time, making it difficult to isolate the role of any single purpose. For example, some digital games can be both educational and entertaining (Budke et al., 2025), which may make it harder for parents to manage media use based solely on purpose. By comparison, screen time and media type may be easier for parents to monitor and regulate and are more likely to become the primary targets of parental regulation.

Collectively, the non-significant findings for use purpose suggest that parental involvement may not be associated with all dimensions of children’s digital media use in the same way. Purpose-based categories are theoretically important, but they may be difficult to separate in everyday family settings. Parents can usually observe how long a child uses digital media and which device is being used. By contrast, the purpose of use may be less visible, more fluid, and more open to interpretation. A single activity may combine learning, entertainment, communication, and emotional regulation, especially among preschool children. This overlap may be one possible explanation for why parental involvement did not significantly moderate the associations between specific purposes of use and parental stress.

Theoretically, parental involvement should not be treated as a general moderator across all aspects of children’s digital media use. Its role may be more relevant for dimensions that are concrete, observable, and easier for parents to regulate, such as screen time and device type. In contrast, the purpose of use may depend more on children’s ongoing engagement and on the mixed nature of digital content.

### 4.4 Implications

This study extends research on preschool children’s digital media use and parental stress by moving beyond screen time alone. Much of the existing literature has treated digital media use as a general exposure, with less attention to the devices children use or the purposes for which they use them. By distinguishing media type and usage purpose, the present study shows that the link between children’s digital media use and parental stress is not uniform. Instead, it varies by screen time, device type, and level of parental involvement.

This study also discusses parental stress from the perspective of parental involvement. The findings suggest that the association between parental involvement and parental stress may depend on the digital context. In some cases, parental involvement may be associated with lower parental stress, which is consistent with previous research (Williams et al., 2020; Hou et al., 2023). However, parents’ involvement with complex emerging digital devices may place an additional cognitive burden and time pressure on them, which may be related to higher parental stress. From a digital parenting perspective, these findings offer a more comprehensive understanding of contextual patterns related to parental stress.

In addition, this study offers practical insights for parents managing children’s digital media use. The findings suggest that parental involvement is not always associated with lower parental stress, and that its role may depend on the type of media involved. Parents may therefore need more differentiated strategies that take into account the features and demands of different digital media.

For traditional media such as television, parents may take a more relaxed approach. Regulation may focus on co-viewing and setting limits on viewing time. For mobile devices such as smartphones and tablets, a more structured approach may be needed because content is more open and use occurs across a wider range of situations. Parents can set clear rules, filter content, and use media together with their children.

Importantly, in the context of emerging digital media, increased parental involvement may be associated with greater cognitive and time demands for parents. These findings suggest that parental involvement is not merely a question of “more is better,” but should also be understood in terms of “appropriateness.” This means that rather than purchasing many new products, parents may focus on a few practical devices. Parents may choose one or two practical digital devices for long-term use, after developing a clear understanding of their features and capabilities.

### 4.5 Limitations and future directions

This study has several limitations. First, although Harman’s single-factor test suggested that common method bias was unlikely to be a major concern, it cannot be completely ruled out because the data were collected from the same respondents using self-report questionnaires at a single time point. Future studies should use multi-informant, observational, or longitudinal designs to reduce potential common method variance.

Second, the method used to measure children’s screen time in this study was relatively simple. Specifically, screen time was assessed using only a single question. Although this approach is common in previous research, it may not fully account for the complexity of young children’s use of digital media (Mallawaarachchi et al., 2022; Seguin et al., 2021). For example, it does not capture differences between weekday and weekend use, or the quality of media content. These features may be relevant to how screen time relates to parental stress, but they could not be examined in the present study. Future research could use more detailed measures, such as separate weekday and weekend estimates, time-use diaries, repeated daily assessments, or digital tracking.

Third, the study had limited control for potential confounding factors. Owing to data access and privacy constraints during questionnaire administration, the moderation models did not fully include child-, parent-, and household-level characteristics. These variables included child age and gender, parent gender and age, parental education, and household socioeconomic status. Kindergarten type was included as a contextual covariate, as it may capture differences in educational resources, family background, and caregiving environments. The observed difference between Kindergarten type 3 and Kindergarten type 1 also indicates that kindergarten type was related to parental stress in this sample. However, kindergarten type is only a contextual indicator and cannot substitute for individual-level demographic or socioeconomic measures. The associations among children’s digital media use, parental involvement, and parental stress may therefore still be partly explained by unmeasured family and demographic factors. Future studies should collect more detailed child-, parent-, and family-level data and include these variables in adjusted models.

In addition, this study used a cross-sectional design. Accordingly, we were unable to infer the direction of causality among the factors. Current research highlights the bidirectional relationship between parental stress and preschool children’s digital media use (Ly et al., 2026). On the one hand, higher levels of parental stress may lead to increased screen time among preschool children (McDaniel and Radesky, 2020; Nandhini et al., 2025). On the other hand, greater screen time may, in turn, contribute to elevated parental stress (Wolfers et al., 2025; Brauchli et al., 2024). A similar bidirectional pattern may also exist between parental involvement and parental stress. As parental stress increases, parents may lower their expectations and reduce their level of involvement (Liu et al., 2024). Conversely, parental involvement can also influence parental stress, with higher levels of involvement potentially helping alleviate it (Luo et al., 2020). These findings suggest that future research should use a longitudinal design to examine the causal relationships among parental involvement, children’s screen time, and parental stress.

Finally, this study did not distinguish between different types of parental involvement. In the context of children’s digital media use, parental involvement is generally conceptualized in terms of several forms, including instructive, co-use, and restrictive mediation (Valkenburg et al., 1999; Livingstone and Helsper, 2008; Shin and Li, 2017). Parental stress may also influence the types of strategies parents adopt. For example, research suggests that when parental stress increases, parents are inclined to rely on instructive mediation to improve children’s ability to understand media content (Warren and Aloia, 2019).

### 4.6 Conclusion

This study examined the role of parental involvement in the association between parental stress and children’s digital media use. The results suggest that the positive association between parental stress and children’s screen time was weaker at higher levels of parental involvement. The positive association between children’s smartphone use and parental stress was also weaker at higher levels of parental involvement. The findings of the present study highlight the important role of parental involvement. Furthermore, the findings highlight the distinct nature of emerging digital media, which showed opposite patterns of association between parental stress and children’s digital media use across different levels of parental involvement. This study extends existing research and offers a more nuanced understanding of how different media environments are associated with family dynamics.

## Data Availability

The raw data supporting the conclusions of this article will be made available by the authors, without undue reservation.

## References

[ref1] Abidin R. R. (1995). Parenting Stress Index: Professional Manual. 3rd Edn Lutz, FL: Psychological Assessment Resources.

[ref2] Aghanyan G. (2023). The role of mobile games in the process of physical education of young school children. Scientific Proceedings of the Vanadzor State University Humanities and Social Sciences. Vanadzor, Armenia: Vanadzor State University. pp. 518–524.

[ref3] Attavar S. P. K. Rani P. (2025). Positive, negative, and ambivalent: Indian parents’ attitudes to and mediation methods of children’s digital media use. Cogent Soci. Sci. 11:2446685.

[ref4] Auxier B. Anderson M. Perrin A. Turner E. (2020) Parenting children in the age of screens Pew Research Center https://www.pewresearch.org/internet/2020/07/28/parenting-children-in-the-age-of-screens/

[ref5] Bandura A. (1977). Self-efficacy: toward a unifying theory of behavioral change. Psychol. Rev. 84, 191–215. doi: 10.1037/0033-295X.84.2.191, 847061

[ref6] Barroso N. E. Mendez L. Graziano P. A. Bagner D. M. (2018). Parenting stress through the lens of different clinical groups: A systematic review and meta-analysis. J. Abnorm. Child Psychol. 46, 449–461. doi: 10.1007/s10802-017-0313-6, 28555335 PMC5725271

[ref7] Bauerlein M. Walesh S. G. (2009). The Dumbest Generation: How the digital age Stupefies young Americans and Jeopardizes our Future. New York, NY: Tarcher/Penguin.

[ref8] Bayar M. E. Kulaksız T. Toran M. (2026). How does parental media mediation regulate the association between digital parental awareness and the parent–child relationship? Early Child. Educ. J. 54, 773–787. doi: 10.1007/s10643-025-01879-x

[ref9] Benedetto L. Ingrassia M. (2021). “Digital parenting: raising and protecting children in media world,” in Parenting: Studies by an Ecocultural and Transactional Perspective, (London: IntechOpen), 127–148.

[ref10] Beneteau E. Boone A. Wu Y. Kientz J. A. Yip J. Hiniker A. (2020) Parenting with Alexa: exploring the introduction of smart speakers on family dynamics. In proceedings of the 2020 CHI conference on human factors in computing systems. New York: Association for Computing Machinery. pp. 1–14

[ref12] Benson P. R. (2015). Longitudinal effects of educational involvement on parent and family functioning among mothers of children with ASD. Res. Autism Spectr. Disord. 11, 42–55. doi: 10.1016/j.rasd.2014.11.011

[ref13] Bentley G. F. Turner K. M. Jago R. (2016). Mothers’ views of their preschool child’s screen-viewing behaviour: A qualitative study. BMC Public Health 16:718. doi: 10.1186/s12889-016-3440-z, 27492488 PMC4973523

[ref9001] Budke A. Czauderna A. Gebele D. Zepter A. L. (2025). Beyond entertainment: analyzing multiperspectivity in digital strategy games for educational purposes. Educ. Sci. 15:479. doi: 10.3390/educsci15040479

[ref9002] Brauchli V. Sticca F. Edelsbrunner P. von Wyl A. Lannen P. (2024). Are screen media the new pacifiers? The role of parenting stress and parental attitudes for children’s screen time in early childhood. Comput. Hum. Behav. 152:108057. doi: 10.1016/j.chb.2023.108057

[ref14] Çalhan C. Göksu İ. (2024). An effort to understand parents’ media mediation roles and early childhood children’s digital game addiction tendency: a descriptive correlational survey study. Educ. Inf. Technol. 29, 17825–17865. doi: 10.1007/s10639-024-12544-y

[ref15] Chaarani B. Ortigara J. Yuan D. Loso H. Potter A. Garavan H. P. (2022). Association of Video Gaming with Cognitive Performance among Children. JAMA Netw. Open 5:e2235721. doi: 10.1001/jamanetworkopen.2022.35721, 36279138 PMC9593235

[ref16] Chaudron S. Beutel M. E. Černikova M. Donoso Navarrete V. Dreier M. Fletcher-Watson B. . (2015). Young Children (0–8) and Digital Technology: A Qualitative Exploratory Study Across Seven Countries. Luxembourg: Publications Office of the European Union.

[ref20] Chong S. C. Teo W. Z. Shorey S. (2023). Exploring parents’ perceptions of children’s screen time: A systematic review and meta-synthesis of qualitative studies. Pediatr. Res. 94, 915–925. doi: 10.1038/s41390-023-02555-9, 36966270 PMC10039437

[ref21] Corkin M. T. Peterson E. R. Henderson A. M. E. Waldie K. E. Reese E. Morton S. M. B. (2021). Preschool screen media exposure, executive functions, and symptoms of inattention/hyperactivity. J. Appl. Dev. Psychol. 73:101237. doi: 10.1016/j.appdev.2020.101237

[ref22] Coyne S. M. Radesky J. S. Collier K. M. Gentile D. A. Linder J. R. Nathanson A. I. . (2017). Parenting and digital media. Pediatrics 140, S112–S116. doi: 10.1542/peds.2016-1758N 29093044

[ref23] Domoff S. E. Borgen A. L. Radesky J. S. Lumeng J. C. Miller A. L. (2019). Excessive use of mobile devices and children’s behavioral problems: the role of parental stress. Child Psychiatry Hum. Dev. 50, 531–542.

[ref24] Fang Y. Luo J. Boele M. Windhorst D. van Grieken A. Raat H. (2024). Parent, child, and situational factors associated with parenting stress: A systematic review. Eur. Child Adolesc. Psychiatry 33, 1687–1705. doi: 10.1007/s00787-023-02171-2, 35876894 PMC11211171

[ref25] Fitzpatrick C. Cristini E. Bernard J. Y. Garon-Carrier G. (2023). Meeting preschool screen time recommendations: which parental strategies matter? Front. Psychol. 14:1287396. doi: 10.3389/fpsyg.2023.1287396, 38022940 PMC10662125

[ref27] French A. N. Ashby R. S. Morgan I. G. Rose K. A. (2013). Time outdoors and the prevention of myopia. Exp. Eye Res. 114, 58–68. doi: 10.1016/j.exer.2013.04.018, 23644222

[ref29] Glatz T. Buchanan C. M. (2015). Over-time associations among parental self-efficacy, promotive parenting practices, and adolescents’ externalizing behaviors. J. Fam. Psychol. 29, 427–437. doi: 10.1037/fam0000076, 25844491

[ref9003] Hayes A. F. (2018). Introduction to Mediation, Moderation, and Conditional Process Analysis: A Regression-Based Approach. 2nd Edn. New York, NY: Guilford Press.

[ref32] Hiniker A. Suh H. Cao S. Kientz J. A. (2016) Not at the dinner table: parents’ and children’s perspectives on family technology rules In Proceedings of the 2016 CHI Conference on Human Factors in Computing Systems New York: Association for Computing Machinery 1376–1389

[ref9004] Hill D. Ameenuddin N. Reid Chassiakos Y. L. Cross C. Hutchinson J. Levine A. . (2016). Media and young minds. Pediatrics 138:e20162591. doi: 10.1542/peds.2016-2591 27940793

[ref33] Holly L. E. Fenley A. R. Kritikos T. K. Merson R. A. Abidin R. R. Langer D. A. (2019). Evidence-Base update for parenting stress measures in clinical samples. J. Clin. Child Adolesc. Psychol. 48, 685–705. doi: 10.1080/15374416.2019.1639515, 31393178

[ref34] Hou Y. Yan T. Zhang J. (2023). The relationship between parental involvement and psychological adjustment among Chinese children with autism spectrum disorder during the transition from kindergarten to primary school: a chain-mediating model. Front. Psychol. 14:1087729. doi: 10.3389/fpsyg.2023.1087729 36891207 PMC9986549

[ref35] Hu B. Y. Johnson G. K. Teo T. Wu Z. (2020). Relationship between screen time and Chinese children’s cognitive and social development. J. Res. Child. Educ. 34, 183–207. doi: 10.1080/02568543.2019.1702600

[ref36] Jing M. Ye T. Kirkorian H. L. Mares M. (2023). Screen media exposure and preschool children’s vocabulary learning and development: A meta-analysis. Child Dev. 94, 1398–1418. doi: 10.1111/cdev.13927, 37042116

[ref37] Johnson G. M. (2010). Internet use and child development: the techno-microsystem. Aust. J. Educ. Dev. Psychol. 10, 32–43.

[ref38] Kattein E. Schmidt H. Witt S. Jörren H. L. Menrath I. Rumpf H.-J. . (2023). Increased digital media use in preschool children: exploring the links with parental stress and problematic media use. Children 10:1921. doi: 10.3390/children10121921 38136123 PMC10742172

[ref39] Kerr M. L. Barr R. Booth M. Blanchard M. A. Suh B. Piper D. J. . (2025). Bidirectional and temporal associations between daily reports of parental burnout, parenting experiences, and motivations for family screen use. Transl. Issues Psychol. Sci. doi: 10.1037/tps0000476, 41178978 PMC12574683

[ref40] Kestler-Peleg M. Lavenda O. Stenger V. Bendett H. Alhalel-Lederman S. Maayan-Metzger A. . (2020). Maternal self-efficacy mediates the association between spousal support and stress among mothers of NICU hospitalized preterm babies. Early Hum. Dev. 146:105077. doi: 10.1016/j.earlhumdev.2020.105077, 32447178

[ref41] Lapierre M. A. Krcmar M. Choi E. Haberkorn K. A. Locke S. J. (2021). The role of family communication in the association between children’s television exposure and family stress. J. Child. Media 15, 362–379.

[ref43] Lee H. E. Kim J. Y. Kim C. (2022). The influence of parent media use, parent attitudes toward media, and parenting style on children’s media use. Children 9:37. doi: 10.3390/children9010037 35053662 PMC8774813

[ref9051] Lazarus R. S. Folkman S. (1984). “Stress: appraisal and coping,” in Encyclopedia of Behavioral medicine, eds. M. D. Gellman and J. R. Turner (Berlin: Springer).

[ref44] Lee A. R. Park Y. W. Oh J. (2023). Investigating the cause and effect factors of young children’s smartphone overuse: focusing on the influence of parenting factors. Inf. Commun. Soc. 26, 1756–1772. doi: 10.1080/1369118X.2022.2027499

[ref45] Lin C. Cui Y. Wang X. Su X. Zhang L. Peng Q. (2025). The impact of parental media attitudes and mediation behaviors on young children’s problematic media use in China: an actor–partner interdependence mediation model analysis. Behav. Sci. 15:1141. doi: 10.3390/bs15081141, 40867497 PMC12382966

[ref46] Liu H. Chen Z. Fang Y. (2024). Association between family screen environment and screen content for preschool children in Shanghai. Chin. J. Sch. Health 45, 421–428. doi: 10.16835/j.cnki.1000-9817.2024255

[ref9005] Lissak G. (2018). Adverse physiological and psychological effects of screen time on children and adolescents: literature review and case study. Environ. Res. 164, 149–157. doi: 10.1016/j.envres.2018.01.015 29499467

[ref47] Livingstone S. Helsper E. J. (2008). Parental mediation of children’s internet use. J. Broadcast. Electron. Media 52, 581–599. doi: 10.1080/08838150802437396

[ref48] Lou J. Wang M. Xie X. Wang F. Zhou X. Lu J. . (2024). The association between family socio-demographic factors, parental mediation and adolescents’ digital literacy: a cross-sectional study. BMC Public Health 24:2932. doi: 10.1186/s12889-024-20284-4, 39438851 PMC11515667

[ref9006] Luo Y. Qi M. Huntsinger C. S. Zhang Q. Xuan X. Wang Y. (2020). Grandparent involvement and preschoolers’ social adjustment in Chinese three-generation families: examining moderating and mediating effects. Child. Youth Serv. Rev. 114:105057. doi: 10.1016/j.childyouth.2020.105057

[ref49] Ly A. Dimitropoulos G. McArthur B. A. Watt J. Cullen E. Madigan S. (2026). A qualitative study examining household context and parental perspectives on preschoolers’ digital media use guidelines. BMC Public Health 26:566. doi: 10.1186/s12889-026-26248-0, 41547835 PMC12895794

[ref50] Madigan S. McArthur B. A. Anhorn C. Eirich R. Christakis D. A. (2020). Associations between screen use and child language skills: a systematic review and meta-analysis. JAMA Pediatr. 174, 665–675. doi: 10.1001/jamapediatrics.2020.0327, 32202633 PMC7091394

[ref51] Mallawaarachchi S. R. Hooley M. Sutherland-Smith W. Horwood S. (2022). “You’re damned if you do, you’re damned if you don’t”: a qualitative exploration of parent motives for provision of mobile screen devices in early childhood. BMC Public Health 22:153. 36324121 10.1186/s12889-022-14459-0 PMC9629764

[ref53] Marsh J. Plowman L. Yamada-Rice D. Bishop J. C. Scott F. Davenport A. . (2016). Exploring play and creativity in pre-schoolers’ use of apps: Final project report. Sheffield: University of Sheffield.

[ref54] Mascheroni G. (2024). A new family member or just another digital interface? Smart speakers in the lives of families with young children. Hum.-Mach. Commun. 7, 45–63. doi: 10.30658/hmc.7.3

[ref55] McDaniel B. T. Radesky J. S. (2020). Longitudinal associations between early childhood externalizing behavior, parenting stress, and child media use. Cyberpsychol. Behav. Soc. Netw. 23, 384–391. 32096655 10.1089/cyber.2019.0478 PMC7301326

[ref56] Nakshine V. S. Thute P. Khatib M. N. Sarkar B. (2022). Increased screen time as a cause of declining physical, psychological health, and sleep patterns: a literature review. Cureus 14:e30317. doi: 10.7759/cureus.30317 36381869 PMC9638701

[ref58] Nikken P. Jansz J. (2014). Developing scales to measure parental mediation of young children’s internet use. Learn. Media Technol. 39, 250–266. doi: 10.1080/17439884.2013.782038

[ref59] Nikken P. Schols M. (2015). How and why parents guide the media use of young children. J. Child Fam. Stud. 24, 3423–3435. doi: 10.1007/s10826-015-0144-4, 26472932 PMC4598347

[ref60] Njoroge W. F. Elenbaas L. M. Garrison M. M. Myaing M. Christakis D. A. (2013). Parental cultural attitudes and beliefs regarding young children and television. JAMA Pediatr. 167, 739–745. doi: 10.1001/jamapediatrics.2013.75, 23778788

[ref61] Operto F. F. Pastorino G. M. G. Scuoppo C. Padovano C. Vivenzio V. Pistola I. . (2021). Adaptive behavior, emotional/behavioral problems, and parental stress in children with autism spectrum disorder. Front. Neurosci. 15:751465. doi: 10.3389/fnins.2021.751465 34899160 PMC8660640

[ref62] Papadakis S. Kalogiannakis M. Zaranis N. (2018a). Educational apps from the Google play store for Greek preschool children: a study on quality and learning value. Interact. Technol. Smart Educ. 15, 55–72.

[ref63] Papadakis S. Kalogiannakis M. Zaranis N. (2018b). Mobile device use among preschool-aged children in Greece. Educ. Inf. Technol. 23, 1–17. 10.1007/s10639-021-10718-6 PMC8406380 34483704

[ref64] Park S. Oh I. (2023). The relationship between parental self-efficacy and parenting stress: a meta-analytic review. Child Youth Serv. Rev. 144:106752. doi: 10.1016/j.childyouth.2022.106752

[ref66] Poitras V. J. Gray C. E. Janssen X. Aubert S. Carson V. Faulkner G. . (2017). Systematic review of the relationships between sedentary behaviour and health indicators in the early years (0–4 years). BMC Public Health 17:868. doi: 10.1186/s12889-017-4849-8, 29219092 PMC5773886

[ref67] Radesky J. S. Kistin C. J. Eisenberg S. Gross J. Block G. Zuckerman B. . (2015). Parent perspectives on their young children’s media use. Pediatrics 136, 1044–1050. doi: 10.1542/peds.2015-2151, 26527548

[ref68] Radesky J. S. Schumacher J. Zuckerman B. (2015). Mobile and interactive media use by young children: the good, the bad, and the unknown. Pediatrics 135, 1–3. doi: 10.1542/peds.2014-2251, 25548323

[ref70] Rezendes D. L. Scarpa A. (2011). Associations between parental anxiety/depression and child behavior problems related to autism spectrum disorders: the roles of parenting stress and parenting self-efficacy. Autism Res. Treat. 2011:395190. doi: 10.1155/2011/395190, 22937246 PMC3420762

[ref71] Nandhini S. Abishek J. R. Kanniappan V. S S. (2025). Parental perceptions of preschooler screen time and physical activity in Chennai. BMC Public Health 25:4335. doi: 10.1186/s12889-025-25527-6 41299515 PMC12751655

[ref72] Şad S. N. Konca A. S. Özer N. Acar F. (2016). Parental e-involvement: a phenomenological research on electronic parental involvement. Int. J. Pedagog. Learn. 11, 163–186. doi: 10.1080/22040552.2016.1227255

[ref73] Sajjan M. (2013). A study of parents’ perceptions about television viewing habits of their children. Adv. Res. J. Soci. Sci. 4, 51–54.

[ref74] Seguin D. Kuenzel E. Morton J. B. Duerden E. G. (2021). School’s out: parenting stress and screen time use in school-age children during the COVID-19 pandemic. J Affect Disord Rep. 6:100217. doi: 10.1016/j.jadr.2021.100217, 34514458 PMC8423665

[ref76] Shetty K. Tewathia N. Bamney U. Rawat V. (2022). Micro- and macro-level economic implications of digital addictions: a case study. MGM J. Med. Sci. 9, 588–592.

[ref77] Shin E. Choi K. Resor J. Smith C. L. (2021). Why do parents use screen media with toddlers? The role of child temperament and parenting stress in early screen use. Infant Behav. Dev. 64:101595. doi: 10.1016/j.infbeh.2021.101595, 34153781

[ref78] Shin W. Li B. (2017). Parental mediation of children’s digital technology use in Singapore. J. Child. Media 11, 1–19. doi: 10.1080/17482798.2016.1203807

[ref9007] Su J. (2025). Kindergarten parents’ perceptions of the use of AI technologies and AI literacy education: positive views but practical concerns. Educ. Inf. Technol. 30, 279–295. doi: 10.1007/s10639-024-12673-4

[ref80] Tay L. Y. Aiyoob T. B. Chua T. B. K. Ramachandran K. Chia M. Y. H. (2021). Pre-schoolers’ use of technology and digital media in Singapore: entertainment indulgence and/or learning engagement? Educ. Media Int. 58, 1–20. doi: 10.1080/09523987.2021.1908498

[ref81] Valkenburg P. M. Krcmar M. Peeters A. L. Marseille N. M. (1999). Developing a scale to assess three styles of television mediation: instructive mediation, restrictive mediation, and social coviewing. J. Broadcast. Electron. Media 43, 52–66. doi: 10.1080/08838159909364474

[ref82] Vedechkina M. Borgonovi F. (2021). A review of evidence on the role of digital technology in shaping attention and cognitive control in children. Front. Psychol. 12:611155. doi: 10.3389/fpsyg.2021.611155, 33716873 PMC7943608

[ref83] Wang J. Wang S. Xiao B. Li J. Feng Y. Li Y. (2024). Maternal distress, parenting stress, maladaptive parenting, and children’s problematic media use in China: evidence from spring 2022 in Shanghai. BMC Public Health 24:1857. doi: 10.1186/s12889-024-19382-0 38992640 PMC11238508

[ref84] Warren R. Aloia L. (2019). Parenting style, parental stress, and mediation of children’s media use. West. J. Commun. 83, 483–500.

[ref85] Warren R. Gerke P. Kelly M. A. (2002). Is there enough time on the clock? Parental involvement and mediation of children’s television viewing. J. Broadcast. Electron. Media 46, 87–111. doi: 10.1207/s15506878jobem4601_6

[ref86] Widyaatmadja S. T. Solechan A. (2024). Digital parenting: challenges and roles of parents in the era of society 5.0. Int. J. Health Med. 1, 165–183. doi: 10.62951/ijhm.v1i3.57

[ref87] Williams K. E. So K. T. Siu T. S. C. (2020). A randomized controlled trial of the effects of parental involvement in supported playgroup on parenting stress and toddler social-communicative behavior. Child Youth Serv. Rev. 118:105364. doi: 10.1016/j.childyouth.2020.105364

[ref88] Wolfers L. N. Nabi R. L. Walter N. (2025). Too much screen time or too much guilt? How child screen time and parental screen guilt affect parental stress and relationship satisfaction. Media Psychology, 28, 102–133. doi: 10.1080/15213269.2024.2310839

[ref90] Wu S. Yu X. Wei W. (2026). The indirect role of children’s screen time and the moderating role of problematic parental screen use on the relationships between different parental mediation strategies and preschoolers’ developmental outcomes. Comput. Educ. 245:105552. doi: 10.1016/j.compedu.2025.105552

[ref91] Yang G.-Y. Huang L.-H. Schmid K. L. Li C.-G. Chen J.-Y. He G.-H. . (2020). Associations between screen exposure in early life and myopia among Chinese preschoolers. Int. J. Environ. Res. Public Health 17:1056. doi: 10.3390/ijerph17031056 32046062 PMC7037286

[ref92] Yao L.-S. Sun X.-J. Niu G.-F. Zheng Y.-L. Chinyani T. (2022). Parental mediation moderates the association between social media exposure and tobacco and alcohol use: differences between elementary and middle school students. J. Stud. Alcohol Drugs 83, 267–275. doi: 10.15288/jsad.2022.83.267, 35254250

[ref93] Yu S. Liao M. Liu X. Li Y. (2025). The relationship between parenting stress and preschool children’s social–emotional competence: the chain mediating role of parental reflective functioning and parent–child relationship. Front. Psychol. 16:1679084. doi: 10.3389/fpsyg.2025.1679084, 41064174 PMC12500748

[ref95] Yu X. Zhang Y. Liu H. Chen J. (2025). Parental reflective functioning and parenting stress: the mediating role of parent–child relationship. Front. Psychol. 16:1679084. 41064174 10.3389/fpsyg.2025.1679084 PMC12500748

[ref9008] Zhang C. (2025). Harsh parenting and preschool children’s screen time: the mediating role of parent–child relationships and the moderating effect of mindful parenting. Front. Psychol. 16:1467701. doi: 10.3389/fpsyg.2025.1467701 40994860 PMC12454304

