# Supplementary material for: Not all digital media use is equal: parental involvement moderates the association between children’s digital media use and parental stress
Source: Front Psychol. 2026 Aug 18;17:1885062. doi: 10.3389/fpsyg.2026.1885062 (PMC13529565; doi:10.3389/fpsyg.2026.1885062)
Supplement: Supplementary file 1 [file Table_1.docx]

**Table S1** Moderation Analysis Predicting Parental Stress: Tablet Use

| **Predictor** | ***B*** | ***SE*** | ***t*** | ***p*** | **LLCI** | **ULCI** |
| --- | --- | --- | --- | --- | --- | --- |
| Constant | 84.550 | 0.808 | 104.609 | < .001 | 82.962 | 86.137 |
| Tablet use (X) | 4.859 | 1.155 | 4.205 | < .001 | 2.590 | 7.128 |
| Parental Involvement(W) | -3.202 | 0.724 | -4.422 | < .001 | -4.624 | -1.780 |
| X × W | -2.643 | 1.444 | -1.830 | .068 | -5.478 | 0.192 |
| Kindergarten type 2 | 1.894 | 1.789 | 1.059 | .290 | -1.619 | 5.407 |
| Kindergarten type 3 | 2.078 | 1.249 | 1.663 | .097 | -0.375 | 4.531 |

Note. X = tablet use; W = parental involvement. Kindergarten type 1 was used as the reference category. HC3 robust standard errors were used. LLCI = lower limit of the 95% confidence interval; ULCI = upper limit of the 95% confidence interval.

**Table S2** Moderation Analysis Predicting Parental Stress: Smartphone Use

| **Predictor** | ***B*** | ***SE*** | ***t*** | ***p*** | **LLCI** | **ULCI** |
| --- | --- | --- | --- | --- | --- | --- |
| Constant | 84.604 | 0.809 | 104.638 | < .001 | 83.016 | 86.192 |
| Smartphone use (X) | 5.637 | 1.174 | 4.801 | < .001 | 3.332 | 7.943 |
| Parental Involvement(W) | -3.082 | 0.709 | -4.346 | < .001 | -4.475 | -1.690 |
| X × W | -3.240 | 1.413 | -2.293 | .022 | -6.015 | -0.465 |
| Kindergarten type 2 | 1.583 | 1.753 | 0.903 | .367 | -1.860 | 5.025 |
| Kindergarten type 3 | 2.614 | 1.286 | 2.032 | .043 | 0.088 | 5.140 |

Note. X = smartphone use; W = parental involvement. Kindergarten type 1 was used as the reference category. HC3 robust standard errors were used. LLCI = lower limit of the 95% confidence interval; ULCI = upper limit of the 95% confidence interval.

**Table S3** Moderation Analysis Predicting Parental Stress: Television Use

| **Predictor** | ***B*** | ***SE*** | ***t*** | ***p*** | **LLCI** | **ULCI** |
| --- | --- | --- | --- | --- | --- | --- |
| Constant | 76.966 | 0.970 | 79.328 | < .001 | 75.061 | 78.871 |
| Televison use (X) | 1.810 | 1.731 | 1.046 | .296 | -1.589 | 5.209 |
| Parental Involvement(W) | -4.002 | 0.930 | -4.304 | < .001 | -5.829 | -2.176 |
| X × W | 1.262 | 2.304 | 0.548 | .584 | -3.262 | 5.785 |
| Kindergarten type 2 | 1.525 | 2.103 | 0.725 | .469 | -2.605 | 5.655 |
| Kindergarten type 3 | 4.156 | 1.551 | 2.680 | .008 | 1.111 | 7.201 |

Note. X = television use; W = parental involvement. Kindergarten type 1 was used as the reference category. HC3 robust standard errors were used. LLCI = lower limit of the 95% confidence interval; ULCI = upper limit of the 95% confidence interval.

**Table S4** Moderation Analysis Predicting Parental Stress: Emerging Digital Device Use

| **Predictor** | ***B*** | ***SE*** | ***t*** | ***p*** | **LLCI** | **ULCI** |
| --- | --- | --- | --- | --- | --- | --- |
| Constant | 76.817 | 0.981 | 78.276 | < .001 | 74.890 | 78.744 |
| Emerging Digital Device Use (X) | -0.186 | 1.660 | -0.112 | .911 | -3.445 | 3.072 |
| Parental Involvement(W) | -3.246 | 0.956 | -3.396 | .001 | -5.123 | -1.369 |
| X × W | 6.119 | 2.192 | 2.792 | .005 | 1.815 | 10.423 |
| Kindergarten type 2 | 1.475 | 2.086 | 0.707 | .480 | -2.621 | 5.571 |
| Kindergarten type 3 | 4.292 | 1.558 | 2.756 | .006 | 1.234 | 7.350 |

Note. X = emerging digital device use; W = parental involvement. Kindergarten type 1 was used as the reference category. HC3 robust standard errors were used. LLCI = lower limit of the 95% confidence interval; ULCI = upper limit of the 95% confidence interval.

**Table sS** Moderation Analysis Predicting Parental Stress: Communication Purpose

| **Predictor** | ***B*** | ***SE*** | ***t*** | ***p*** | **LLCI** | **ULCI** |
| --- | --- | --- | --- | --- | --- | --- |
| Constant | 84.324 | 0.828 | 101.875 | < .001 | 82.698 | 85.949 |
| Communication Purpose  (X) | -1.438 | 1.177 | -1.222 | .222 | -3.749 | 0.872 |
| Parental Involvement(W) | -2.992 | 0.830 | -3.604 | < .001 | -4.622 | -1.362 |
| X × W | -0.368 | 1.746 | -0.211 | .833 | -3.796 | 3.060 |
| Kindergarten type 2 | 1.861 | 1.780 | 1.046 | .296 | -1.634 | 5.355 |
| Kindergarten type 3 | 2.745 | 1.263 | 2.173 | .030 | 0.264 | 5.225 |

Note. X = communication use; W = parental involvement. Kindergarten type 1 was used as the reference category. HC3 robust standard errors were used. LLCI = lower limit of the 95% confidence interval; ULCI = upper limit of the 95% confidence interval.

**Table S6**Moderation Analysis Predicting Parental Stress: Emotional Regulation Purpose

| **Predictor** | ***B*** | ***SE*** | ***t*** | ***p*** | **LLCI** | **ULCI** |
| --- | --- | --- | --- | --- | --- | --- |
| Constant | 84.497 | 0.820 | 103.030 | < .001 | 82.887 | 86.108 |
| Emotional Regulation Purpose  (X) | 4.573 | 1.423 | 3.214 | .001 | 1.779 | 7.366 |
| Parental Involvement(W) | -3.097 | 0.776 | -3.990 | < .001 | -4.621 | -1.573 |
| X × W | -1.703 | 1.809 | -0.941 | .347 | -5.255 | 1.850 |
| Kindergarten type 2 | 1.487 | 1.791 | 0.830 | .407 | -2.030 | 5.003 |
| Kindergarten type 3 | 2.381 | 1.261 | 1.888 | .060 | -0.096 | 4.858 |

Note. X = emotional regulation use; W = parental involvement. Kindergarten type 1 was used as the reference category. HC3 robust standard errors were used. LLCI = lower limit of the 95% confidence interval; ULCI = upper limit of the 95% confidence interval.

**Table S7**Moderation Analysis Predicting Parental Stress: Educational Purpose

| **Predictor** | ***B*** | ***SE*** | ***t*** | ***p*** | **LLCI** | **ULCI** |
| --- | --- | --- | --- | --- | --- | --- |
| Constant | 84.295 | 0.834 | 101.093 | < .001 | 82.658 | 85.933 |
| Educational Purpose  (X) | -1.083 | 1.283 | -0.844 | .399 | -3.601 | 1.436 |
| Parental Involvement(W) | -2.962 | 0.816 | -3.631 | < .001 | -4.563 | -1.360 |
| X × W | 0.994 | 1.605 | 0.619 | .536 | -2.158 | 4.146 |
| Kindergarten type 2 | 1.901 | 1.813 | 1.048 | .295 | -1.659 | 5.461 |
| Kindergarten type 3 | 2.714 | 1.265 | 2.145 | .032 | 0.230 | 5.198 |

Note. X = educational use; W = parental involvement. Kindergarten type 1 was used as the reference category. HC3 robust standard errors were used. LLCI = lower limit of the 95% confidence interval; ULCI = upper limit of the 95% confidence interval.

**Table S8**Moderation Analysis Predicting Parental Stress: Entertainment Purpose

| **Predictor** | ***B*** | ***SE*** | ***t*** | ***p*** | **LLCI** | **ULCI** |
| --- | --- | --- | --- | --- | --- | --- |
| Constant | 84.311 | 0.821 | 102.715 | < .001 | 82.699 | 85.923 |
| Entertainment Purpose  (X) | 1.610 | 1.442 | 1.116 | .265 | -1.222 | 4.441 |
| Parental Involvement(W) | -2.983 | 0.817 | -3.650 | < .001 | -4.588 | -1.378 |
| X × W | -0.906 | 1.798 | -0.504 | .615 | -4.437 | 2.626 |
| Kindergarten type 2 | 1.851 | 1.782 | 1.039 | .299 | -1.649 | 5.351 |
| Kindergarten type 3 | 2.705 | 1.271 | 2.129 | .034 | 0.210 | 5.200 |

Note. X = entertainment use; W = parental involvement. Kindergarten type 1 was used as the reference category. HC3 robust standard errors were used. LLCI = lower limit of the 95% confidence interval; ULCI = upper limit of the 95% confidence interval.
